# A potential role for interleukin-33 and γ-epithelium sodium channel in the pathogenesis of human malaria associated lung injury

**DOI:** 10.1186/s12936-015-0922-x

**Published:** 2015-10-05

**Authors:** Sumate Ampawong, Urai Chaisri, Parnpen Viriyavejakul, Panote Prapansilp, Georges E. Grau, Gareth D. H. Turner, Emsri Pongponratn

**Affiliations:** Department of Tropical Pathology, Faculty of Tropical Medicine, Mahidol University, Bangkok, Thailand; Vascular Immunology Unit, Department of Pathology, Sydney Medical School, The University of Sydney, Parramatta Road, Camperdown, NSW Australia; Mahidol-Oxford Tropical Medicine Research Unit (MORU), Faculty of Tropical Medicine, Mahidol University, Bangkok, Thailand; Nuffield Department of Clinical Medicine, Centre for Tropical Medicine and Global Health, Oxford University, Oxford, UK

**Keywords:** Aquaporin, Gamma-epithelial sodium channel, Interleukin-33, Leukocyte, Malaria, Pathogenesis, *Plasmodium falciparum*, Pulmonary oedema

## Abstract

**Background:**

The pathogenesis of pulmonary oedema (PE) in patients with severe malaria is still unclear. It has been hypothesized that lung injury depends, in addition to microvascular obstruction, on an increased pulmonary capillary pressure and altered alveolar-capillary membrane permeability, causing pulmonary fluid accumulation.

**Methods:**

This study compared the histopathological features of lung injury in Southeast Asian patients (n = 43) who died from severe *Plasmodium falciparum* malaria, and correlated these with clinical history in groups with or without PE. To investigate the expression of mediators that may influence fluid accumulation in PE, immunohistochemistry and image analysis were performed on controls and sub-sets of patient with or without PE.

**Results:**

The expression of leukocyte sub-set antigens, bronchial interleukin (IL)-33, γ-epithelium sodium channel (ENaC), aquaporin (AQP)-1 and -5, and control cytokeratin staining was quantified in the lung tissue of severe malaria patients. Bronchial IL-33 expression was significantly increased in severe malaria patients with PE. Malaria patients with shock showed significantly increased bronchial IL-33 compare to other clinical manifestations. Bronchial IL-33 levels were positively correlated with CD68+ monocyte and elastase + neutrophil, septal congestion and hyaline membrane formation. Moreover, the expression of both vascular smooth muscle cell (VSMC) and bronchial γ-ENaC significantly decreased in severe malaria patients with PE. Both VSMC and bronchial γ-ENaC were negatively correlated with the degree of parasitized erythrocyte sequestration, alveolar thickness, alveolar expansion score, septal congestion score, and malarial pigment score. In contrast AQP-1 and -5 and pan cytokeratin levels were similar between groups.

**Conclusions:**

The results suggest that IL-33 may play a role in lung injury during severe malaria and lead to PE. Both VSMC and bronchial γ-ENaC downregulation may explain pulmonary fluid disturbances and participate in PE pathogenesis in severe malaria patients.

## Background

Malaria infection caused by *Plasmodium falciparum* continues to be a major cause of death in tropical countries. Death in falciparum malaria is a consequence of multiple vital organ dysfunction, including cerebral malaria (CM), anaemia, acidosis, renal failure, and respiratory insufficiency [1]. The degree and type of lung involvement varies between cases, some patients show no involvement or minor symptoms such as cough, other show chest X-ray (CXR) changes or develop respiratory distress, or progress to frank pulmonary oedema or acute respiratory distress syndrome (ARDS), the cause of death in adults with severe malaria-associated lung injury [2–4]. Hospital-based case series of severe malaria have reported clinical development of pulmonary oedema (PE) in 9–23 % of patients [5–7]. Autopsy studies [8, 9] in patients dying from severe falciparum malaria have revealed heavy oedematous lungs, congested pulmonary capillaries, thickened alveolar septa, intra-alveolar haemorrhages, hyaline membrane formation, and serous pleural as well as pericardial effusions. Microscopically, in addition to alveolar oedema and haemorrhages, the alveolar capillaries are filled with sequestrated *P. falciparum*-infected red blood cells and host leukocytes, such as malarial pigment-laden macrophages. Clinical and pathological evidence suggests that this is a non-cardiogenic form of PE [10–12]. In some series pulmonary involvement is as common as coma [13, 14].

The pathophysiology of impaired lung function in falciparum malaria is poorly understood, but has been studied in murine models of experimental severe malaria. It is likely to involve both pathogen-induced changes such as pulmonary microvascular obstruction by parasitized red blood cells (PRBCs) [15], malaria pigment associated lung injury [16] and host factors, such as soluble cytokine release [17] and leukocyte responses. Other groups have hypothesized that lung injury following treatment of malaria may be predominantly an inflammatory response, rather than purely a microvascular obstructive phenomenon, as evidenced by abnormal gas transfer and lung symptoms in both uncomplicated (mild) falciparum malaria, and lung injury in other malarias such as *Plasmodium vivax* [18].

Although lung pathology has been a relatively neglected area in the study of malaria complications [19], understanding the pathophysiology must account for the range of symptoms seen in patients. Where frank acute PE develops, this is may be a poor prognosis sign and a terminal event, possibly reflecting brainstem injury in comatose patients and neurogenic in origin. In patients with ‘algid’ malaria, characterized by cardiogenic shock, oedema may result from aggressive fluid resuscitation. However a lesser degree of PE, and other lung infiltrates on CXR are common, which argues for a direct pulmonary pathogenesis in some cases [3].

In the normal lung, fluid moves from the blood circulation through the capillary endothelium into the lung interstitium and then is cleared by the lymphatics on a continuous basis [20]. When the permeability of capillary walls is increased, as in acute lung injury (ALI), the quantity of fluid that leaves the pulmonary microcirculation is increased to a point that overwhelms the clearance capacity of the lymphatics leading to interstitial oedema (hydrostatic oedema) [21]. However, eventually alveolar oedema (permeability oedema) will develop if the amount of interstitial oedema overwhelms the epithelial barrier and overflows into the airspaces [20]. Clearance of PE fluid is accomplished by active ion transport, especially by the alveolar epithelium [20]. The molecular mechanisms through which oedema formation is regulated in the lung are complex. The current paradigm for liquid homeostasis in the adult mammalian lung is that passive apical uptake of sodium via the amiloride-sensitive epithelial Na^+^ channel (ENaC) creates the major driving force for re-absorption of water through the alveolar epithelium in addition to other ion channels, such as potassium and chloride channels [22]. ENaC can be expressed on both bronchial epithelium but also vascular smooth muscle cell (VSMC) in bronchial walls. Other water transport membrane proteins may also have a role. To date, 13 different aquaporins (AQPs) have been identified in mammalian cells [23]. In the respiratory system, AQP-1 is localized in microvascular endothelial cells and AQP-5 in the apical side of epithelium cells [24, 25]. Recent evidence has suggested a role for AQP-1 and -5 in maintenance of fluid homeostasis in the lung [25–29], and some alterations due to tissue damage may lead to pathophysiologic conditions [30–33].

In experimental model, sepsis induced acute lung injury, Interleukin (IL)-33 deficient mice exhibit reduced mortality and cytokine release [34]. IL-33 is a newly characterized member of the IL-1 superfamily of cytokines that is mainly expressed by epithelial cells, and its expression is upregulated following pro-inflammatory stimulation. It is thought to function as an ‘alarmin’, released following cell necrosis to alert the immune system to tissue damage or stress [35]. IL-33 also is involved in T cell-mediated immune responses and functions as a central regulator of human auto-immune diseases [36].

In this study, the histopathological features of lung involvement in 43 adult Thai patients dying of severe falciparum malaria were examined in post-mortem lung samples and ten trauma cases as normal controls. Sub-sets of malaria patients either with or without histological evidence of PE were then examined using immunohistochemistry to investigate leukocyte sub-set localization (including CD3, CD8, CD68, and neutrophil elastase), the expression of bronchial IL-33, γ-ENaC, AQP-1 and -5, and pancytokeratin as a control. Histopathological features such as septal thickness and immunolocalization of staining were further analysed using digital image analysis. The correlation between the expression of these markers, histopathological and clinical parameters was evaluated to determine whether IL-33, any distinct subtypes of host leukocytes, γ-ENaC, and AQP-1 and -5 were associated with the development of PE in severe malaria lung injury.

## Methods

### Ethical permission

This research protocol was approved by the Ethics Committee of the Faculty of Tropical Medicine, Mahidol University, Thailand (approval number MUTM2012-032-01). The Ethics Committee waived the need for consent since this study was the leftover specimen study. All relevant clinical data were permitted and obtained from Hospital for Tropical Diseases, Thailand and analysed anonymously.

### Sample collection

This study used formalin-fixed, paraffin-embedded specimens of lung tissue previously collected at post mortem from patients who died from severe falciparum malaria at Hospital for Tropical Diseases, Thailand, between 1969 and 2004. Lung specimens were fixed in 10 % formalin at autopsy and processed using standard histological techniques, and stored as paraffin-embedded blocks. All patients had careful admission clinical examination and investigations. On admission, the presence of a sexual *P. falciparum* blood stages in the peripheral blood was detected by routine parasitological examination all patients. Severe malaria was classified according to WHO criteria as the presence of one or more of the following complications: a Glasgow Coma score of less than 11 (CM, anaemia, jaundice, renal failure, PE, hypoglycaemia, shock, and hyperparasitaemia) [37]. As well as severe falciparum malaria, some patients had evidence of either clinical pneumonia (increased respiratory rate + intercostal indrawing with or without consolisation on CXR).

### Histological study

Histopathological examination was conducted on standard 4 micron thickness, haematoxylin and eosin (H&E)-stained slides of the malaria lung (n = 43 including non-PE = 15, PE = 28). These were assessed for the presence of PE, alveolar wall expansion, septal congestion, PRBC sequestration, malarial pigment, intra-alveolar haemorrhage, hyaline membrane formation, and accumulation of leukocytes (WBC). Septal congestion was determined by the increasing of alveolar thickness with cells including WBC, red blood cell (RBC), and PRBC, while alveolar wall expansion was categorized by its expansion with increased alveolar epithelium cells and/or hyaline membrane formation without cellular congestion. Cases were defined as showing evidence of PE if more than 25 % of the alveolar spaces in the section contained free fluid. The overall histopathological grade of each feature was classified as: 0 = absent, +1 = focal (less than 25 % of section), +2 = present (between 25 and 50 % of section) and +3 = severe (more than 50 % of section). These morphological parameters were compared with normal lung tissue from controls, consisting of trauma cases (n = 10) with no gross pathological changes at autopsy or on subsequent histological examination of the lung. Histopathological parameters were fully blind-graded by two observers (SA and GT). A third person (EP) was asked for additional grading when more than 2 score was observed. Complete clinical data were available in 31/43 cases (11 non-PE and 20 cases with PE).

### Immunohistochemistry study

Nine markers, all T-cells (CD3), cytotoxic T cell (CD8), monocyte/macrophage (CD68), neutrophil elastase, IL-33, γ-ENaC, AQP-1 and -5, and pan cytokeratin (AE1/AE3), were used as described in Table 1. Four micron thick sections from the paraffin blocks were cut and placed on precoated immunohistochemistry slides, dried overnight at 56 °C then allowed to cool. The sections were deparaffinized in xylene and rehydrated prior to immunostaining.

**Table 1:** Primary antibodies used in the study

Antibodies	Type	Source	Dilution	Measuring site
CD3	Mouse monoclonal	DakoCytomation^®^	1:100	All T-lymphocyte
CD8	Mouse monoclonal	DakoCytomation^®^	1:100	Cytotoxic T-lymphocyte
CD68	Mouse monoclonal	DakoCytomation^®^	1:200	Macrophage
Neutrophil elastase	Mouse monoclonal	DakoCytomation^®^	1:200	Neutrophil
IL-33	Rabbit polyclonal	Milipore^®^	1:100	Bronchial epithelium
γ-ENaC	Rabbit polyclonal	Milipore^®^	1:100	Bronchial epithelium Vascular smooth muscle cell
AQP-1	Rabbit polyclonal	Milipore^®^	1:100	Alveolar capillary
AQP-5	Rabbit polyclonal	Milipore^®^	1:50	Alveolar epithelium
Pan cytokeratin	Rabbit polyclonal	Milipore^®^	1:400	Bronchial epithelium

Heat-induced antigen retrieval with citrate buffer, pH 6 or EDTA Tris buffer, pH 9 was used to unmask the antigen depending on the optimized method for each antibody, according to manufacturer’s instructions. Endogenous peroxidase was quenched with 3 % v/v hydrogen peroxide in methanol after sections were cooled. Sections were washed with 0.2 % v/v Tween in phosphate buffered saline (0.2 % T-PBS) and blocked with protein block serum free (Dako, Denmark, X0909) for 10 min. Sections were incubated primary antibody diluted in PBS with 1 % v/v normal goat serum (NGS, Vector, USA, S1000), washed three times in 0.2 % T-PBS and incubated for 30 min with labelled polymer HRP antimouse/rabbit EnVision kit (Dako, Denmark, K5007) at room temperature, according to manufacturer instruction, and visualized with diaminobenzidine (DAB, DAKO, Denmark, K3468) for 3 min, then the sections washed and counterstained with haematoxylin and permanently mounted with DPX.

### Quantitative assessment of leukocyte immunostaining

The number of leukocytes in the lung sections was quantitated by the number of immunopositive cells counted in ten randomly selected high power (×400) fields. The criteria for definite positive cell staining were cytoplasmic or membrane staining with the relevant antibody in a section where the nucleus of the particular cell can be seen.

### Assessment of bronchial immunohistochemistry for bronchial IL-33, pan cytokeratin, and γ-ENaC using digital image analysis

In each group, five random fields (at ×400) were examined for all observed bronchi. From each specimen, colour images of 640 × 480 pixel resolution were acquired with a light microscope (BX51, Olympus^®^) and digital camera (DP20, Olympus^®^). Immunohistochemical expression was then be analysed by semi-quantitative digitalized image analysis using ImageJ, NIH^®^ [38]. Briefly, colour images were first converted to 8-bit image in grey scale. Adjusted images were transformed by threshold mode to locate the interested area. The polygon selection mode was used to outline the area of bronchus. Then, the area of positive immunostaining was estimated by the number of black pixels within measuring mode. Thus, the area fraction of positive reaction was determined as the percentage of black pixels/bronchus area.

### Image analysis for assessment of alveolar thickness

In each case, multiple random fields (at ×400) were examined to assess the thickness of the alveolar septum. Ten random colour images of 640 × 480 pixel resolution were acquired as above. Alveolar thickness was measured (5 thickness/image) by straight-line selection mode after length calibration by set scale mode using the image analysis software ImageJ, NIH^®^.

### Assessment of arterial immunohistochemistry for VSMC γ-ENaC

In each case, multiple random fields (at ×400) were examined for ten arteries. Colour images were acquired, and then the expression area of VSMC γ-ENaC was analysed as previously described using the tunica media as the denominator measurement.

### Assessment of alveolar immunohistochemistry for AQP-1 and -5

Ten random fields (at ×400) were examined for alveolar septa. From each specimen, colour images were acquired, and the expression area of AQP-1 staining on alveolar vessels and AQP-5 staining on alveolar epithelium was analysed as previously mentioned using total alveolar vessel wall or septal length, respectively, as a denominator measurement.

### Statistical analysis

Data were statistically analysed with IBM^®^ SPSS^®^ Statistic software version 20. Kruskal-Wallis test was used to compare area of expression of AQP-1 and -5, pancytokeratin, γ-ENaC and IL-33 among normal, PE and non-PE lungs. Spearman’s correlation test was used to determine the correlation between measured parameters. The proportion of each clinical complication was compared between PE and non-PE groups by Chi-square test. Histopathological score and number of leukocyte sub-sets were compared by each clinical complication using Mann–Whitney U test. The criterion for significance was considered as p value less than 0.05.

## Results

### Clinical data

Clinical manifestations and parameters in severe malaria patients are described in Table 2. The clinical signs of PE in adults did not differ from that of younger patients, [75.0 % (15/20) and 45.5 % (5/11), respectively, p = 0.132]. The age, parasitaemia on admission and percentage of PRBC sequestration were not different between non-PE and PE patients. There was no difference in patterns of clinical manifestations of severe malaria between non-PE and PE patients, apart from anaemia and acidosis, which were significantly more frequent in non-PE patients (p = 0.012 and 0.013, respectively).

**Table 2:** Clinical data, histopathological changes and clinical complications of severe malaria cases

Parameter	Non-PE	PE	p value
Clinical data and complication			
Age (years)	5–42 (20.8 ± 14.6)	3–54 (24.5 ± 13.4)	0.485
Parasitaemia/µl on admission	412,042.72 ± 224,608.34	594,576.50 ± 822,975.64	0.480
% PRBC sequestration	65.5 ± 40.68	74.0 ± 36.18	0.552
Anaemia (%)	100 (11/11)	55 (11/20)	0.012
Jaundice (%)	54.5 (6/11)	80 (16/20)	0.217
Pneumonia (%)	45.5 (5/11)	45 (9/20)	1.000
Acute renal failure (%)	27.3 (3/11)	30 (6/20)	1.000
Acidosis (%)	63.6 (7/11)	15 (3/20)	0.013
Shock (%)	9.1 (1/11)	20 (4/20)	0.631
Systemic bleeding (%)	9.1 (1/11)	20 (4/20)	0.631
Hypoglycaemia (%)	18.2 (2/11)	5 (1/20)	0.281
Cerebral malaria (%)	63.6 (7/11)	70 (14/20)	1.000
Histopathological changes (median score)			
Alveolar expansion	1.46 ± 1.24	1.64 ± 0.95	0.607
Septal congestion	1.73 ± 1.09	1.78 ± 0.68	0.848
Malarial pigment	1.73 ± 1.09	2.00 ± 0.86	0.385
Alveolar haemorrhage	0.93 ± 1.09	1.03 ± 1.13	0.778
PRBCs sequestration	1.46 ± 1.12	1.42 ± 0.95	0.908
WBC accumulation	2.20 ± 0.77	2.00 ± 0.60	0.356
Hyaline membrane formation	0.13 ± 0.35	0.39 ± 0.73	0.037
Treatments (number/cases)			
Quinine	3/11	11/20	–
Artemeter	0/11	2/20	–
Quinine and artesunate	0/11	1/20	–
Artesunate and artemeter	1/11	0/20	–
Data were not available	7/11	6/20	–

### Histopathology

**Fig. 1:**
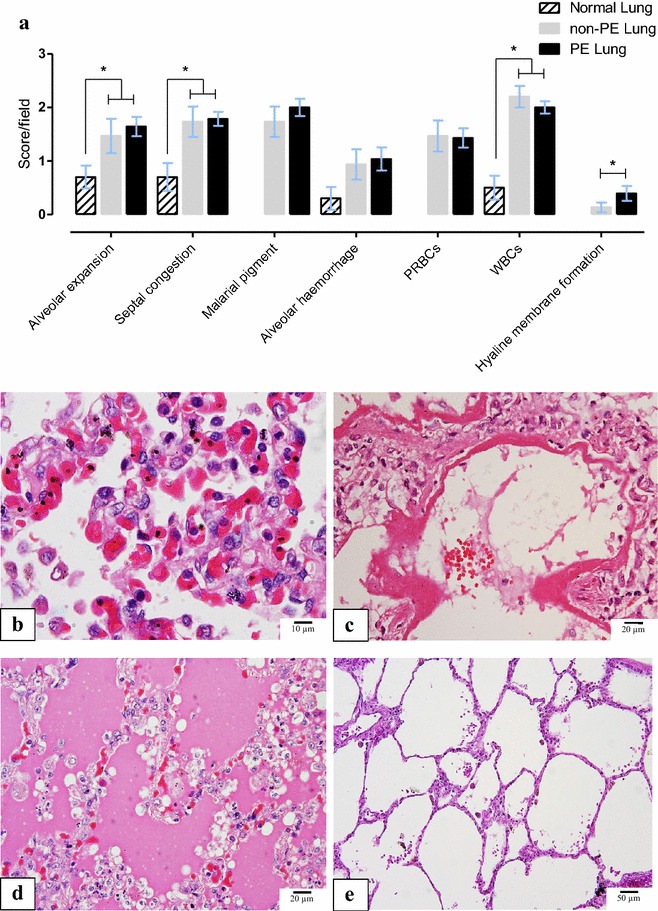
Histopathological changes in the lung in severe malaria. **a** *Bar chart* comparing score of the histopathological features in normal, non-PE and PE malaria lung samples; **b**, **c** photomicrographs of histopathological changes in a fatal falciparum malaria case [case number A41001]; **b** alveolar capillary expansion with accumulation of sequestration PRBCs and leukocytes accumulation. Phagocytosed haemozoin pigment in macrophages and neutrophils within alveolar septa; **c** pulmonary oedema and hyaline membrance formation indicated severe lung injury; **d** severe pulmonary oedema presented with free oedematous fluid in alveolar space [case number A47001-8]; and, **e** normal lung with clear alveolar space and thin alveolar septum [case number RC002-22], (H&E staining, *scale bars* as shown), For this and subsequent figures statistical significance of comparisons are *p < 0.05, **p < 0.01, ***p < 0.0001

Lung sections from patients with severe malaria showed PRBC sequestration, alveolar wall expansion, septal congestion, accumulation of malarial pigment-laden macrophages, neutrophils, and other WBCs, intra-alveolar haemorrhages, and hyaline membrane formation. Of 43 patients with severe malaria enrolled in the study, 28 (~65 %) had evidence of PE. Histopathological scores among PE, non-PE, and normal lungs are showed in Fig. 1. All parameters exhibited significantly lower scores in normal lung. The scores were not different between PE and non-PE except for hyaline membrane formation, which was significantly higher in PE than in non-PE and control lungs. The occurrence of hyaline membrane formation, indicating diffuse alveolar damage (and correlating with the clinical development of ARDS) did not differ between adult patients (≥18 years old) and paediatric patients (<18 years old), [35.0 % (7/20) and 36.3 % (4/11), respectively, p = 0.675]. Image analysis revealed that alveolar septal thickness was not significantly different among normal, PE, and non-PE lungs (17.41 ± 9.20, 23.29 ± 10.14 and 20.97 ± 22.11, respectively, p = 0.747).

### Bronchial IL-33

**Fig. 2:**
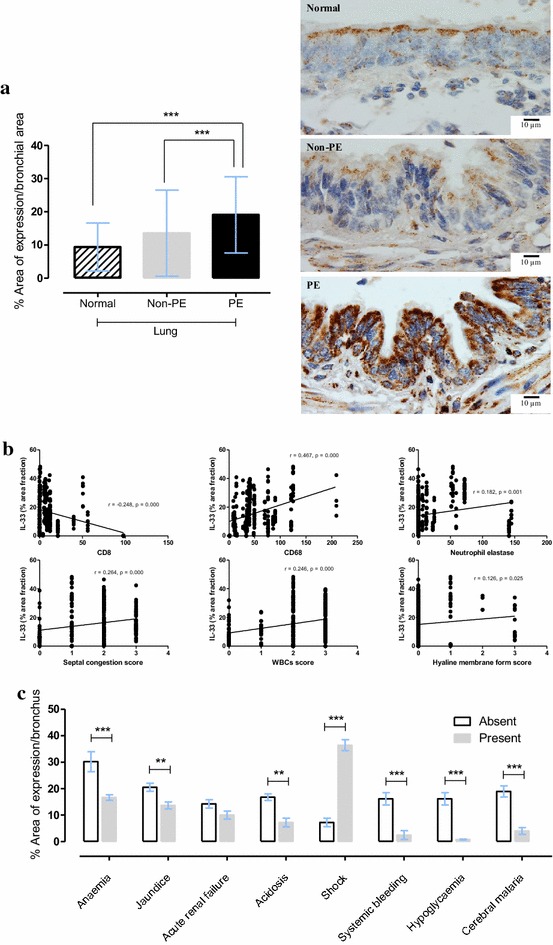
Immunohistochemical expression of bronchial IL-33 expression is increased in malaria cases with pulmonary oedem. **a** *Bar chart* comparing of bronchial IL-33 expression in normal, non-PE and PE lung samples and photomicrographs of immunohistochemical staining of bronchial IL-33 localization; **b** significance correlation plot of bronchial IL-33 expression to leukocyte count and histopathological score; **c** *bar chart* comparing of bronchial IL-33 expression to clinical manifestations

Bronchial IL-33 expression was significantly higher in cases with PE than in the non-PE and normal lungs (Fig. 2a). While there was a significantly negative correlation between bronchial IL-33 expression and CD8+ T-cell accumulation (number/hpf), a significantly positive correlation was found between bronchial IL-33 expression and CD68+ monocyte accumulation (number/hpf), elastase + neutrophils (number/hpf), septal congestion score, total leukocyte score, and hyaline membrane formation score (Fig. 2b). Moreover, the patients who showed co-existent clinical signs of shock in severe malaria exhibited significantly higher bronchial IL-33 expression (Fig. 2c).

### Leukocyte sub-set accumulation

**Fig. 3:**
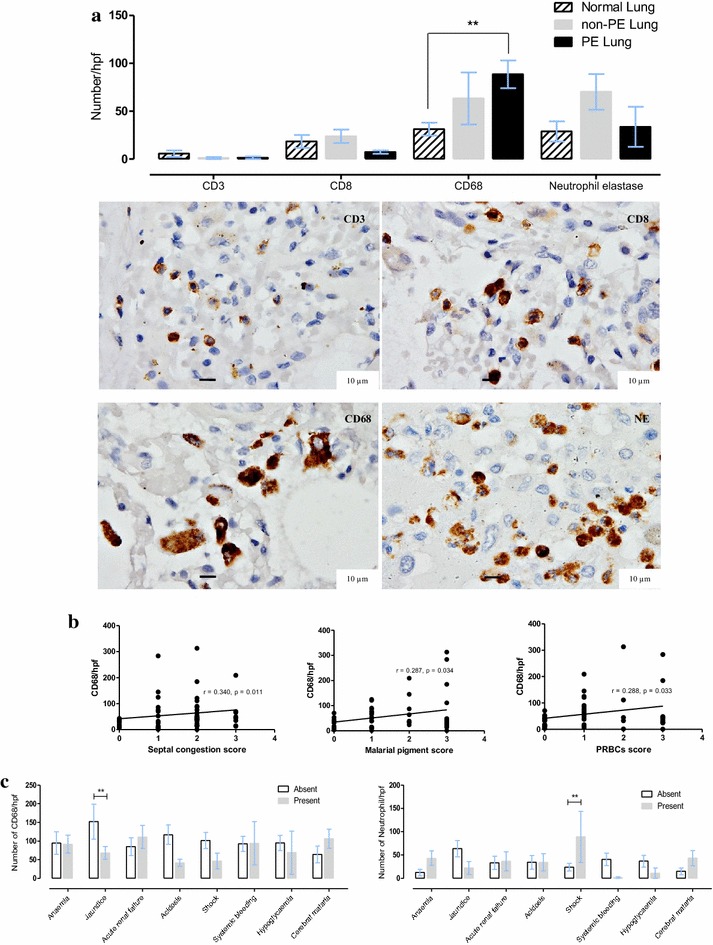
Alveolar leukocyte sub-set counts compared between clinical groups and histopathological features. **a** *Bar chart* comparing of leukocyte sub-sets count in normal, non-PE and PE lung samples and photomicrographs of immunohistochemical staining of leukocyte sub-sets localization; **b** significance correlation plot of leukocyte sub-sets count to histopathological score; **c** significance *bar chart* comparing of leukocyte sub-sets count to clinical manifestations

The numbers of CD3, CD8, CD68 cells, and neutrophils were not different among non-PE, PE, and normal lungs (Fig. 3a). However a significantly positive correlation between CD68 and septal congestion score, malarial pigment score and PRBCs score was found (Fig. 3b). The patients who showed the clinical signs of shock also exhibited significantly higher numbers of neutrophils while there was no difference in leukocyte subsets in relation with these signs (Fig. 3c). Also, the number of neutrophils was not different between patients with (16.62 ± 24.95) and without (49.20 ± 101.58) hyaline membrane formation.

### γ-ENaC

**Fig. 4:**
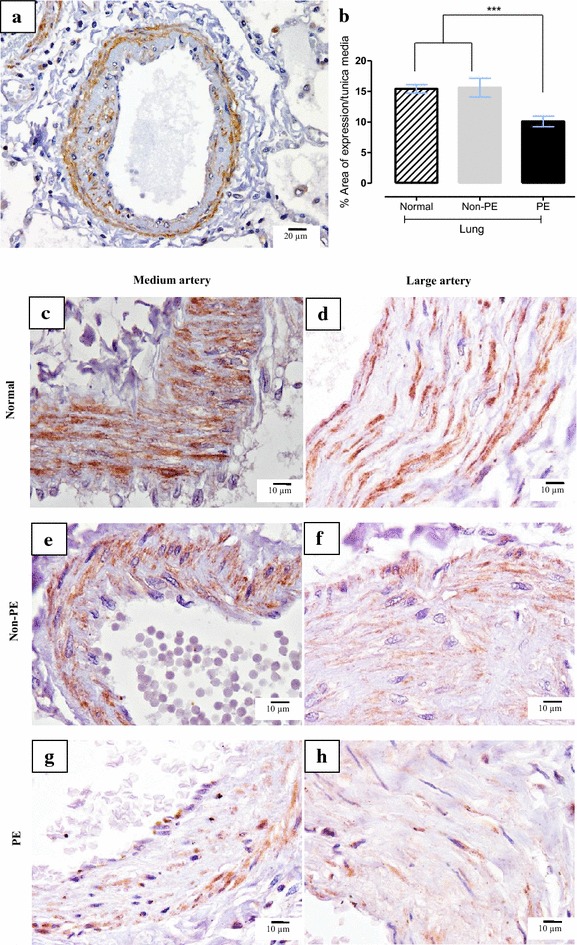
Expression of vascular smooth muscle cell (VSMC) γ-ENaC. **a** Photomicrograph of small arteries tunica media’s VSMC show positive cytoplasmic immunostaining pattern; **b** *Bar chart* comparing of VSMC γ-ENaC expression in normal, non-PE and PE lung samples; photomicrographs of immunohistochemical staining of VSMC γ-ENaC in both medium-size and large arteries in normal (**c**, **d**), non-PE (**e**, **f**) and PE (**g**, **h**) lung samples

**Fig. 5:**
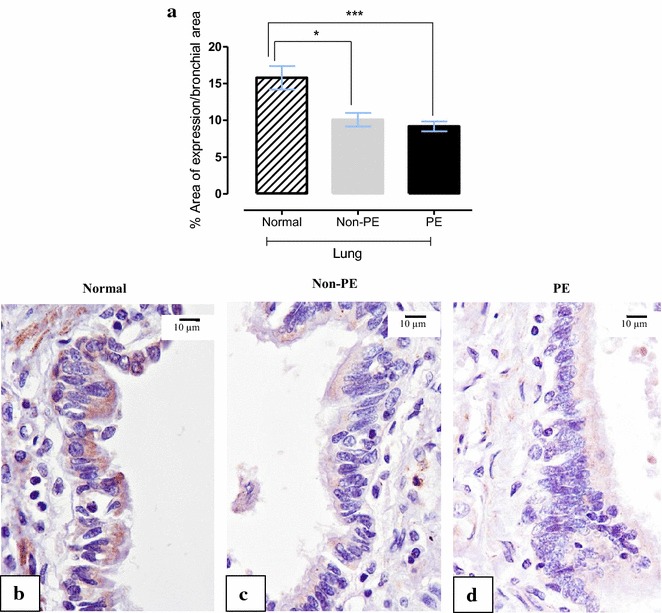
Immunohistochemical staining for bronchial γ-ENaC. **a** *Bar chart* comparing of bronchial γ-ENaC expression in normal, non-PE and PE lung samples; photomicrographs of immunohistochemical staining of bronchial γ-ENaC localization in normal (**b**), non-PE (**c**) and PE (**d**) lung samples

ENaC staining was seen both on bronchial epithelium and vascular smooth muscle cell. Percentage expression areas of VSMC γ-ENaC were significantly lower in PE than in non-PE and normal lungs (Fig. 4) while the expression of bronchial γ-ENaC in both non-PE and PE were significantly lower than normal lungs (Fig. 5). The results also indicate a significantly negative correlation between both VSMC and bronchial γ-ENaC to alveolar thickness (−0.251; p < 0.001 and −0.349; p < 0.001, respectively), alveolar expansion score (−0.141; p < 0.05 and −0.306; p < 0.001, respectively), septal congestion score (−0.141; p < 0.05 and −0.257; p < 0.01, respectively), and malarial pigment score (−0.336; p < 0.001 and −0.196; p < 0.05, respectively). Moreover, the patients who showed clinical signs of anaemia (10.03 ± 5.53 compared to 14.13 ± 8.38, p < 0.01) and pneumonia (9.01 ± 5.96 compared to 13.26 ± 7.59, p < 0.05) had significantly decreased levels of bronchial γ-ENaC.

### AQP-1 and -5 and cytokeratin

**Fig. 6:**
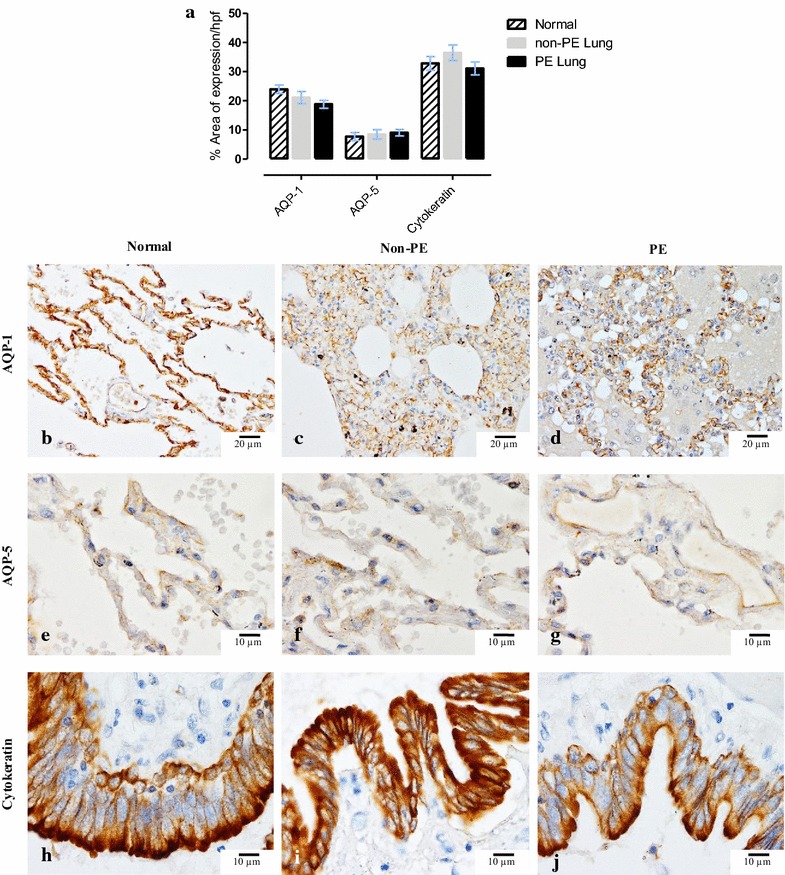
Immunohistochemical staining for AQP-1 and -5 and bronchial cytokeratin. **a** *Bar chart* comparing the degree of alveolar expression of AQP-1 and -5 and bronchial expression of cytokeratin in normal, non-PE, and PE-lung samples; photomicrographs of immunohistochemical staining of AQP-1 and -5 (localized on alveolar vessel and alveolar epithelium, respectively) and bronchial cytokeratin among normal (**b**, **e**, **h**), non-PE (**c**, **f**, **i**) and PE (**d**, **g**, **j**) lung samples

AQP-1 stained endothelial cell, AQP-5 staining pattern on alveolar/bronchial epithelial cells. These patterns are similar to those shown in previous publications. The expression levels of alveolar AQP-1 and -5 scoring and bronchial cytokeratin were not significantly different between severe malaria patients with or without PE and healthy subjects (Fig. 6). The maintenance of staining for pancytokeratin across groups allowed comparison of changes in other markers, given the potential for loss of immunoreactivity in blocks stored for some years.

## Discussion

Among clinical parameters, no manifestations of severe falciparum malaria were associated with a higher incidence of PE, whilst only features which were associated with lower incidence of PE were anaemia and acidosis. Peripheral parasitaemia on admission did not differ between PE and non-PE patients, suggesting that multi-organ dysfunction in this group of patients was not related to greater parasite burden, in agreement with the study of Gachot et al. [13]. Quantitation of PRBC sequestration in the lung is a better correlate of tissue parasite burden but again this was not significantly different between cases with or without PE. CM and metabolic acidosis are the most common manifestations of severe malaria in all age groups [39]. Severe anaemia, hypoglycaemia and convulsions are more common in semi-immune paediatric cases (typically in sub-Saharan Africa), whereas renal failure, severe jaundice, ALI, and ARDS are more frequent in adults [2, 3]. The present data showed a similar distribution of clinical manifestations in both adults and children (ranging from three to 54 years old), but confirms the clinical and pathological occurrence of ALI in paediatric cases of severe malaria in Thailand. However, all of the specimens included in this study were historical samples, all clinical details were particularly not complete to obtain. Although anti-malarial treatment was provided in Table 2, there were no available results of blood culture examination and supportive treatments history, such as pulmonary ventilation. These are the study’s limitation since septicaemia and tracheal intubation might also be a cause of PE and lowered γ-ENaC [40].

Pathological features of lung injury in the different groups were heterogeneous. Although PE in severe malaria is characteristically patchy histologically, and we only examined one to two blocks per case, clinical findings and a threshold pathological score (25 % of the section showing free fluid in the alveolar space) were used as important criteria to differentiate PE and non-PE in this study. Some patients dying of malaria show minimal reaction even to high levels of PRBC and WBC sequestration, through PE to pneumonitis-like leukocyte infiltration of alveolar capillaries (which may show up on CXR as patchy infiltrates and be misdiagnosed as PE) to full blown ARDS. Ten patients in this study showed evidence of hyaline membrane formation evidence of early diffuse alveolar damage (DAD), which is correlated with the clinical development of ARDS. PE is likely to be an early event and could be influenced by direct adhesive signalling events resulting from sequestration of PRBC and WBC, and immune-inflammatory network activation in reaction to that in alveolar capillaries. However other factors such as disturbance of epithelial function may also be important, hence examining IL-33, sodium channel and AQPs.

Bronchial expression of IL-33 was significantly increased in patients with PE compared to those without PE and healthy subjects. Furthermore, a positive correlation was found between bronchial IL-33 expression and the number of CD68 + monocytes and neutrophils, septal congestion score, WBC score, and hyaline membrane formation score, suggesting IL-33 may play-role in lung destruction in severe malaria. Some studies showed that IL-33 may be elaborated T cells tracking to an inflammatory site, served as chemo-attractant for human T helper (Th)2 cells, a crucial cytokine for Th2-mediated host response, and a central role in epithelial cells controlling immune responses [41, 42]. It is able to activate cells of both the innate and adaptive immune system, and depending on the disease can either promote the resolution of inflammation or drive disease pathology [35]. According to previous studies, falciparum malaria infection is characterized by elevation of Th2 cytokines expression [43, 44]. However the mechanism by which IL-33 is involved in lung injury in falciparum malaria remains unclear, thorough analysis of either Th1 and Th2 cytokines or Th cell sub-sets with their relevant transcription factors contributing to the level of bronchial epithelial IL-33 still needs to be further studied.

In the lung, IL-33 is mainly expressed in bronchial epithelial cells [45]. Thus, airway epithelial cells are also active players in the pathogenesis of lung malaria through epithelial cytokines, including IL-33 produced in response to various exogenous stimuli or to cellular damage [46]. IL-33 is elevated in patients with pulmonary diseases [35, 47]. A recent report on IL-33 transgenic mice showed pulmonary inflammation with a significant accumulation of lymphocytes, monocytes and neutrophils but mild accumulation of eosinophils in the lung [47, 48]. This observation may correspond to a result of the significant positive correlation between bronchial IL-33 and CD68, neutrophil and WBCs score. Although the results showed a negative correlation between bronchial IL-33 and CD8 T cells, in *P. falciparum* infection, CD8 + cytotoxic T lymphocytes were significantly reduced indicating that immune suppression is more pronounced [44].

Patients with severe malaria due to *P. falciparum*, and more rarely due to *P. vivax* and *Plasmodium knowlesi* may develop ALI and ARDS, often several days after anti-malarial drug treatment [2, 3]. The result demonstrated a significant positive correlation between bronchial IL-33 and hyaline membrane formation, WBC and neutrophils together with a significantly higher incidence of hyaline membrane formation in PE than non-PE. These findings are in agreement with the notion that ARDS pathophysiology centres on inflammatory-mediated increased capillary permeability or endothelial damage, leading to DAD that can continue after parasite clearance [2].

Although this study demonstrated a similar range of leukocyte sub-sets (CD3, CD8, CD68, and neutrophil) among PE, non-PE and healthy subjects, the total score of WBC accumulation was significantly higher in severe malaria patients compared to normal cases. This supports the concept of excessive accumulation of WBCs in the lung as a feature contributing to severe malaria ALI. Interestingly, the correlation between bronchial IL-33 and WBCs was found as previously described. These also appeared in a previous study on paediatric CM [49] which indicated that the number of pulmonary T-cells (as measured by CD3, CD4 or CD8) and pulmonary macrophages (CD68) were similar across CM, non-CM, and non-malarial diagnosed cases.

The results demonstrate that the VSMC expression of γ-ENaC was significantly decreased in patients with PE compared with non-PE and healthy subjects. Moreover, bronchial expression of γ-ENaC was significantly decreased in patients with and without PE when compared to healthy subjects. Due to the age of some of the cases, and prolonged storage, a constitutive antigen was examined to compare the potential loss of antigenicity in sections. However AE1/AE3 is a board spectrum pancytokeratin stain and quantitatively showed no differences in lung of patients with or without PE, and healthy subjects. Similarly, the expression of AQP-1 and -5 showed no significant differences amongst malarial patients with or without PE, or between malaria cases and controls.

There was a significant negative correlation between VSMC and bronchial γ-ENaC expression and the percentage of PRBC sequestration, alveolar thickness, alveolar expansion score, septal congestion score, and malarial pigment score, suggesting that γ-ENaC may play an important role in mediating clearance of oedema fluid from the lung in severe malaria.

The importance of γ-ENaC protein in VSMC-mediated, pressure-induced constriction in arteries has been demonstrated [50, 51]. The myogenic response is an important regulatory mechanism for blood flow autoregulation to the circulation [52]. The reduction of VSMC γ-ENaC leads to decreased myogenic constriction and consequently to increased pressure transmission to the delicate microvasculature and increased susceptibility to end organ injury [53]. In the current study, VSMC γ-ENaC was significantly decreased in patients with PE. Lung injury in PE may result from an increased lung microvascular pressure due to downregulation of VSMC γ-ENaC, leading to an increase permeability of the gas-blood barrier, which is likely to contribute to the formation or maintenance of either interstitial or alveolar PE.

The bronchial circulation also plays a significant role in the formation and re-absorption of both hydrostatic and permeability oedema [54] mediated by various ion pumps and channels such as AQPs, cystic fibrosis transmembrane conductance regulator (CFTR), ENaC, and cyclic nucleotide-gated (CNG) [55]. Severe malaria patients with PE had a reduced bronchial γ-ENaC expression. In addition, the low expression level of both VSMC and bronchial γ-ENaC was correlated to high percentage of alveolar vascular sequestration of PRBC and high malarial pigment scores, implying a high tissue parasite burden in these patients. The mechanisms by which pathogens affect ENaC expression are still being elucidated; C57BL/6 mice infected with *Plasmodium berghei* K173 (PbK) demonstrated a reduction of activity and expression of ENaC suggesting that the infectious agents can have direct effects on ENaC in the lung epithelium [19]. These results imply that falciparum malaria causes a down regulation of γ-ENaC contributing to PE.

Hypoxia has been shown to reduce ENaC activity [56]. The results demonstrate that severe malaria patients who presented anaemia and pneumonia had reduced expression of bronchial γ-ENaC, and these clinical complications can lead to hypoxemic condition and alveolar hypoxia, respectively.

In severe malaria patients, no difference was seen in the expression of AQP-1 and -5 between controls and malaria cases either with or without PE. This is in agreement with many reports showing that AQP-1 and -5 does not appear to play a role in the physiological clearance of oedema in the lung or in the accumulation of extravascular water in the injured lung [27–29, 57]. These studies suggest that AQP-1 and -5 only provide a pathway for osmotically induced water transport across the pleural barrier and do not affect pleural fluid dynamics in the endothelial cell injury.

These results suggest that induced bronchial IL-33 expression may be involved in lung injury and the genesis of PE in severe malaria ALI. Moreover, the reduction both VSMC and bronchial γ-ENaC may retard fluid clearance from the lung and lead to PE in severe malaria. Alveolar AQP-1 and -5 did not show changes in expression, so do not appear to play a major role in deregulated water movement in malaria-induced lung injury. Results from this study identify a potential casual relationship between γ-ENaC and lung oedema pathogenesis in severe malaria. However other aspects require further study, for example the mechanism of channel gating by either mechanical stimuli or extrinsic factors. Not all patients with severe malaria develop lung injury and in this study, histopathological features were broadly similar between groups exhibiting PE or not, hence more detailed research is required to determine the factors which lead to PE or the development of more severe ARDS in individual patients.

## Conclusions

Bronchial expression of the cytokine IL-33 is increased in malaria patients with oedema, may be involved in lung injury and the pathogenesis of oedema in severe malaria. Moreover, reduced levels of the epithelial sodium channel γ-ENaC but not AQP-1 and -5, were seen, which may retard fluid clearance from the lung and contribute to oedema in severe malaria.
